# Case Report: Cambodian National Malaria Surveillance Program Detection of *Plasmodium knowlesi*

**DOI:** 10.4269/ajtmh.22-0039

**Published:** 2022-07-13

**Authors:** Christina Yek, Sreyngim Lay, Jennifer A. Bohl, Somnang Man, Sophana Chea, Chanthap Lon, Vida Ahyong, Cristina M. Tato, Joseph L. DeRisi, Siv Sovannaroth, Jessica E. Manning

**Affiliations:** ^1^ Department of Critical Care Medicine, National Institutes of Health Clinical Center, Bethesda, Maryland;; ^2^ Laboratory of Malaria and Vector Research, National Institute of Allergy and Infectious Diseases, National Institutes of Health, Rockville, Maryland;; ^3^ International Center of Excellence in Research, National Institute of Allergy and Infectious Diseases, National Institutes of Health, Phnom Penh, Cambodia;; ^4^ Vaccine Research Center, National Institute of Allergy and Infectious Diseases, National Institutes of Health, Bethesda, Maryland;; ^5^ National Center of Parasitology, Entomology, and Malaria Control, Phnom Penh, Cambodia;; ^6^ Chan Zuckerberg Biohub, San Francisco, California

## Abstract

Despite recent success in reducing the regional incidence of *Plasmodium falciparum* malaria, cases of zoonotic malaria are on the rise in Southeast Asia. The Cambodian National Malaria Surveillance Program has previously relied on rapid diagnostic tests and blood smear microscopy with confirmatory polymerase chain reaction (PCR) testing in a subset of cases to further distinguish *P. falciparum, P. malariae, P. ovale,* and *P. vivax* species. Here, metagenomic next-generation sequencing identified *P. knowlesi* mono-infection in six Cambodian patients initially diagnosed with *P. malariae* by blood smear microscopy in February–May 2020. These findings of recent human infections with *P. knowlesi* in Cambodia led to the incorporation of *P. knowlesi*–specific PCR diagnostics to national malaria surveillance efforts.

## INTRODUCTION

In May 2015, the WHO approved the Global Technical Strategy for Malaria (GTS) with the overarching goal of reducing the global malaria burden by 90% in 2030. The Greater Mekong subregion (GMS), encompassing Cambodia, China, Laos, Myanmar, Thailand, and Vietnam, was identified as an area of particular interest given the prevalence of multidrug-resistant strains of *Plasmodium falciparum* along the Cambodia–Thailand border.^1^ Public policy in the GMS has concentrated on the elimination of *P. falciparum*; in the 2010s, the Mekong Malaria Elimination Program introduced multifaceted measures including robust surveillance systems, outreach to rural and migrant populations, vector control, bed net distribution, and early combination treatment to address possible drug-resistant strains.^2^ Ongoing deforestation is also contributing to malaria case reduction given shrinking habitats for anopheline mosquito vectors, typically thought to be zoophilic, outdoor feeders that reside primarily in the forest or forest fringe.^3^ The recently published 2020 WHO World Malaria Report highlighted the interim success of regional efforts, with malaria cases in the GMS falling by 90% between 2000 and 2019 and cases of *P. falciparum* malaria falling by 97%, meeting GTS interim milestones.^1^

While elimination efforts for *P. falciparum* appear to be on track, reports of zoonotic malaria are emerging with increasing frequency in the GMS and surrounding countries.^4^^–^^6^ In Malaysia, where molecular diagnosis is part of national malaria surveillance and enables accurate species reporting, all indigenous malaria cases observed between 2018 and 2020 were attributed to *P. knowlesi*, and incidence appears to be increasing (1,600 to over 4,000 cases from 2016 to 2018).^1^ In contrast, Cambodia, which constitutes approximately 58% of all malaria cases in the GMS, has relatively few reports of zoonotic malaria. It is not clear which vectors primarily contribute to ongoing residual malaria transmission given Cambodia’s great diversity of known and cryptic vectors, but these are likely *Anopheles *spp. that predominate in the forest.^7^ One survey conducted in malaria clinics in Pailin Province over a decade ago identified *P. knowlesi* in two symptomatic Cambodian patients.^8^ In 2015, a malariometric survey in Pailin and Battambang provinces found 8 asymptomatic cases of *P. knowlesi* and 11 asymptomatic cases of *P. cynomolgi*.^9^ National surveillance of malaria in Cambodia is conducted by the National Center for Parasitology, Entomology, and Malaria Control, a central entity that collates data submitted by regional government-run healthcare facilities on monthly malaria cases.^10^ Cases are diagnosed by rapid diagnostic tests or blood microscopy, with a subset undergoing polymerase chain reaction (PCR) testing to confirm species classification (*P. falciparum, P. vivax, P. ovale,* and *P. malariae).* In the absence of routine national surveillance for *P. knowlesi* species, the true prevalence of disease in Cambodia remains unknown.

## IDENTIFICATION OF CASES OF *P. KNOWLESI*

During February–May 2020, 966 symptomatic individuals were screened for malaria at Tasanh Health Center in Battambang Province as part of national surveillance conducted by the Cambodian National Malaria Control Program. Of these, 12 cases of suspected malaria had negative rapid diagnostic testing (SD FK80 p.f/P.v Malaria Antigen Rapid Test, Standard Diagnostics, Korea) but detectable parasites on blood smear microscopy; morphology appeared consistent with *P. malariae*. A dried blood spot from the index case (patient P1; Table 1) was submitted for metagenomic next-generation sequencing (mNGS) to identify the parasite. The DNA was extracted from the dried blood spot and used for mNGS library preparation and sequencing on an iSeq100 (Illumina) platform. Sequencing data was stripped for host reads and aligned to NCBI nucleotide and protein databases with IDseq, a cloud-based, open-source bioinformatics platform, as previously described.^11^ *Plasmodium knowlesi* was identified and attributed to 20% of the reads that could not be mapped to the human genome. All putative *P. knowlesi* reads were realigned by BLAST to the NCBI collection of *Plasmodium* sequences, and in all cases, *P. knowlesi* remained the best alignment. After this finding, a multiplex real-time PCR assay was modified to include primers specific to *P. knowlesi* (RealStar Malaria PCR kit, Altona Diagnostics, Germany) and used to evaluate samples from the remaining 11 patients. Five additional cases of *P. knowlesi* were detected by PCR and confirmed using mNGS (patients P2–P6). The remaining six samples were positive for *P. malariae* on PCR. All samples analyzed by mNGS and PCR were de-identified before processing. All patients received a combination regimen of artesunate and mefloquine along with a single low dose (7.5 mg) of primaquine according to national malaria treatment guidelines. Per the NIH Office of Human Subjects Research Protections’ policy under the revised Common Rule, this research does not qualify as human subject research because it used de-identified patient samples from a national surveillance program to sequence pathogens.

**Table 1:** Case description of six patients with *Plasmodium knowlesi*—Battambang Province, Cambodia (February–May 2020)

Patient	Age	Gender	Temperature at presentation (°C)	mNGS rPM*	PK RT-PCR CT†
P1	38	M	37.5	2200	Not performed
P2	26	M	38.0	49	29
P3	36	M	38.0	1758	26
P4	39	M	37.9	3091	24
P5	25	M	37.0	59	30
P6	25	M	Unknown	3269	24

mNGS = metagenomic next-generation sequencing; PCR = polymerase chain reaction; rPM = reads per million.

* Metagenomic next-generation sequencing reads per million of *Plasmodium knowlesi* hits.

†* Plasmodium knowlesi* real-time PCR cycle threshold.

## DISCUSSION

This study describes six cases of *P. knowlesi* in Cambodian patients presenting with acute febrile illness, all of whom had initially been diagnosed as having *P. malariae* based on blood smear microscopy. An accurate diagnosis was achieved retrospectively with mNGS and real-time PCR. All patients received oral combination therapy targeting artemisinin-resistant parasites, which is also active against nonsevere *P. knowlesi*, though follow-up data was not available to assess clinical outcomes.

An early distinction of *P. knowlesi* from other forms of malaria is crucial in guiding clinical management including initial triage, targeted therapy, and subsequent monitoring. Although its disease course is generally benign, *P. knowlesi* has been associated with a risk of severe disease that potentially exceeds that of *P. falciparum* (odds ratio [OR] 2.96, 95% CI 1.19–7.38 in one case series).^12^ In addition, its erythrocytic cycle of approximately 24 hours is shorter than that of human malarial species (48–72 hours), which leads to potentially more rapid clinical progression if left untreated.^13^ However, available diagnostic methods are limited. While blood smear microscopy is widely available, it has low sensitivity and is often unable to distinguish between the morphologically similar early trophozoites of *P. knowlesi* and *P. falciparum,* and the mature band-form trophozoites, schizonts, and gametocytes of *P. knowlesi* and *P. malariae*.^14^ Rapid serologic tests have been the mainstay of rural malarial surveillance efforts, but commercial assays demonstrate suboptimal performance for detecting *P. knowlesi*.^15^ Polymerase chain reaction with species-specific targets remains the definitive diagnostic method, but most malaria-endemic countries in Southeast Asia have yet to incorporate this into national surveillance.^16^

The lack of integrated *P. knowlesi*–specific surveillance has stymied efforts to understand the distribution of the pathogen in the GMS.^16^ Small-range geographical surveys have mapped *P. knowlesi* cases to the fringes of forested regions where the convergence of human hosts, wildlife reservoirs (e.g., macaques), and vectors (*Anopheles *spp.) prime for increased transmission of *P. knowlesi*.^17^^,^^18^ One study applied probability mapping to classify at-risk regions and found several areas of Indonesia, the Philippines, Cambodia, Myanmar, and Vietnam to have high disease potential, but unbiased geo-surveillance data from these countries is limited.^19^

Changing land use, in particular deforestation for subsistence farming or industry, has been associated with an increased risk of zoonotic disease transmission; however, most malaria elimination programs fail to address such practices. Vector control measures are mostly directed at endophagic vectors with the use of indoor insecticide spraying and insecticide-lined bednets, with limited efficacy against the predominantly exophagic vectors of zoonotic malaria. Finally, the gradual elimination of nonzoonotic malaria species from the human population may be contributing to a loss of endemic immunity that could play a role in cross-protection against zoonotic species.^20^ These factors may have contributed to the rise of *P. knowlesi* in Malaysia, where nonzoonotic malaria has otherwise been eradicated in recent years. The case of Malaysia serves as a cautionary tale for malaria-endemic countries in the region, which need to consider integrating the detection of zoonotic malaria into existing surveillance frameworks.

Recognizing the public health importance of zoonotic malaria cases, the Cambodian National Malaria Surveillance Program incorporated *P. knowlesi*–specific PCR to diagnostics in 2021. More work remains to be done including improving geo-surveillance of parasites, vectors, and host reservoirs; developing point-of-care diagnostic assays; describing disease pathogenesis, treatment, and outcomes; incorporating targeted efforts at zoonosis control in malaria elimination efforts; and promoting disease awareness.

Although cases of human malaria may be declining, these findings of recent infections with *P. knowlesi* in Cambodia are a reminder of the increasing importance of zoonotic malaria in malaria-endemic countries. It may be necessary to revise existing surveillance systems to monitor cases of zoonotic malaria and account for these before certifying countries free of malaria, so as to truly meet WHO malaria elimination goals.
